# Small brains predisposed Late Quaternary mammals to extinction

**DOI:** 10.1038/s41598-022-07327-9

**Published:** 2022-03-31

**Authors:** Jacob Dembitzer, Silvia Castiglione, Pasquale Raia, Shai Meiri

**Affiliations:** 1 School of Zoology, Tel Aviv University, 6997801 Tel Aviv, Israel; 2 Dipartimento di Scienze della Terra, dell’Ambiente e delle Risorse, Università di Napoli Federico II, 80126 Naples, Italy; 3 The Steinhardt Museum of Natural History, Tel Aviv University, 6997801 Tel Aviv, Israel

**Keywords:** Evolution, Zoology, Ecology

## Abstract

The Late Quaternary witnessed a dramatic wave of large mammal extinctions, that are usually attributed to either human hunting or climatic change. We hypothesized that the large mammals that survived the extinctions might have been endowed with larger brain sizes than their relatives, which could have conferred enhanced behavioral plasticity and the ability to cope with the rapidly changing Late Quaternary environmental conditions. We assembled data on brain sizes of 291 extant mammal species plus 50 more that went extinct during the Late Quaternary. Using logistic, and mixed effect models, and controlling for phylogeny and body mass, we found that large brains were associated with higher probability to survive the Late Quaternary extinctions, and that extant species have brains that are, on average, 53% larger when accounting for order as a random effect, and 83% when fitting a single regression line. Moreover, we found that models that used brain size in addition to body size predicted extinction status better than models that used only body size. We propose that possessing a large brain was an important, yet so far neglected characteristic of surviving megafauna species.

## Introduction

The Late Quaternary (~ 115 ka–500 years ago) is marked by a drastic extinction event, mainly of large-bodied land-vertebrates (‘megafauna’^1–3^). The main causes put forth to explain the extinction crisis are the rapid climate changes that took place at that time (especially from the last glacial maximum until the end of the last glacial period ~ 25–12 ka), changing species habitats, and overhunting by humans that started to expand across the globe^4–8^. Animals that had never encountered humans before may have been more likely to succumb to extinction even under low hunting pressure^9,10^. In keeping with this, the extinction wave was more intense in the continents humans colonized for the first time, that is in the Americas and Oceania^7^. It has also been proposed that species with traits that make them less prone to human hunting (arboreal, nocturnal, or forest dwelling) were more likely to survive^4,11^ (but see Ref.^12^). Conversely, the onset of fully glacial conditions at 26 ka, and the rapid deglaciation which took place in the 16 to 12 ka interval, may have reduced the available habitat space for most species, driving some to extinction^13,14^. A combined effect of climate change and human hunting have also been put forward as responsible for the extinction wave^15,16^.

Regardless the extinction driver, one prominent feature shared by many extinct taxa was their large body size. In mammals, body size is correlated with several traits, including low population density, small population size, long lifespans, long gestation periods and inter-birth intervals, and low fecundity^17,18^, that are said to enhance extinction risk, under even low levels of population decline^11,19,20^. Brain size is tightly correlated with body size as well^21,22^, and yet, mammals of similar size can have greatly different brain sizes. In extant birds and mammals, relatively large brains have been found to improve survivability (via behavioral flexibility) to novel environments and threats^23–25^. Such behavioral flexibility could have been crucial during the Late Quaternary when species were facing rapidly changing climates and a novel predator in the form of expanding human populations. For example, large-brained animals could more successfully remember the locations of pasture and water sources^26,27^ and respond better to the threat of hunting by humans^9,25,28,29^. It has been hypothesized that Thylacines (*Thylacinus cynocephalus*) and Tasmanian devils (*Sarcophilus harrisii*) were outcompeted by dingoes on mainland Australia, partially due to their larger brains^30,31^. However, large brains have also been associated with long gestation periods and interbirth intervals^32,33^, which were, in turn, linked to increased vulnerability to extinction^34,35^. These findings indicate that the role, if any, of relatively large brains on the chance for survival in megafauna species is still uncertain but worth being investigated.

Here we aimed to: (1) Test whether there is a difference in brain size between extant species and those that went extinct during the Late Quaternary, after the effects of body size and phylogeny are accounted for, in order to understand whether extant species have larger or smaller brains, and by how much. This was analyzed with regression models also accounting for phylogeny. (2) Test whether brain size is a significant correlate of Late Quaternary probability of extinction above and beyond the effects of body size alone using Phylogenetic Generalized Linear Mixed Models and accounting for potential differences in brain size allometric relationships among mammalian orders^36^. We calculated brain size evolutionary rates using the RRphylo package in R^37^ and analyzed our data using phylogenetic trees that assume a Brownian Motion model of evolution, and trees that were rescaled according to RRphylo rates. This allows us to establish whether large brains might have enabled otherwise extinction-prone species to survive the Late Quaternary.

## Results

We recorded brain endocast volume, and body mass, for 50 extinct and 291 extant mammal species based on 3616 specimens (1–411 specimens per species, median = 3; Supplementary material), belonging to 10 taxonomic orders all of which, except Notoungulata, have both extinct and extant members^38^. Body masses ranged from 1.4 to 3850 kg and 11 to 11,000 kg in extant and extinct species, respectively. Xenarthrans, Proboscideans, and Primates, made up half of the dataset, which is a good reflection of which species went extinct during the Late Quaternary^7^. We used mean body mass from the literature for 18 species and this did not affect our results (Supplementary material).

**Figure 1:**
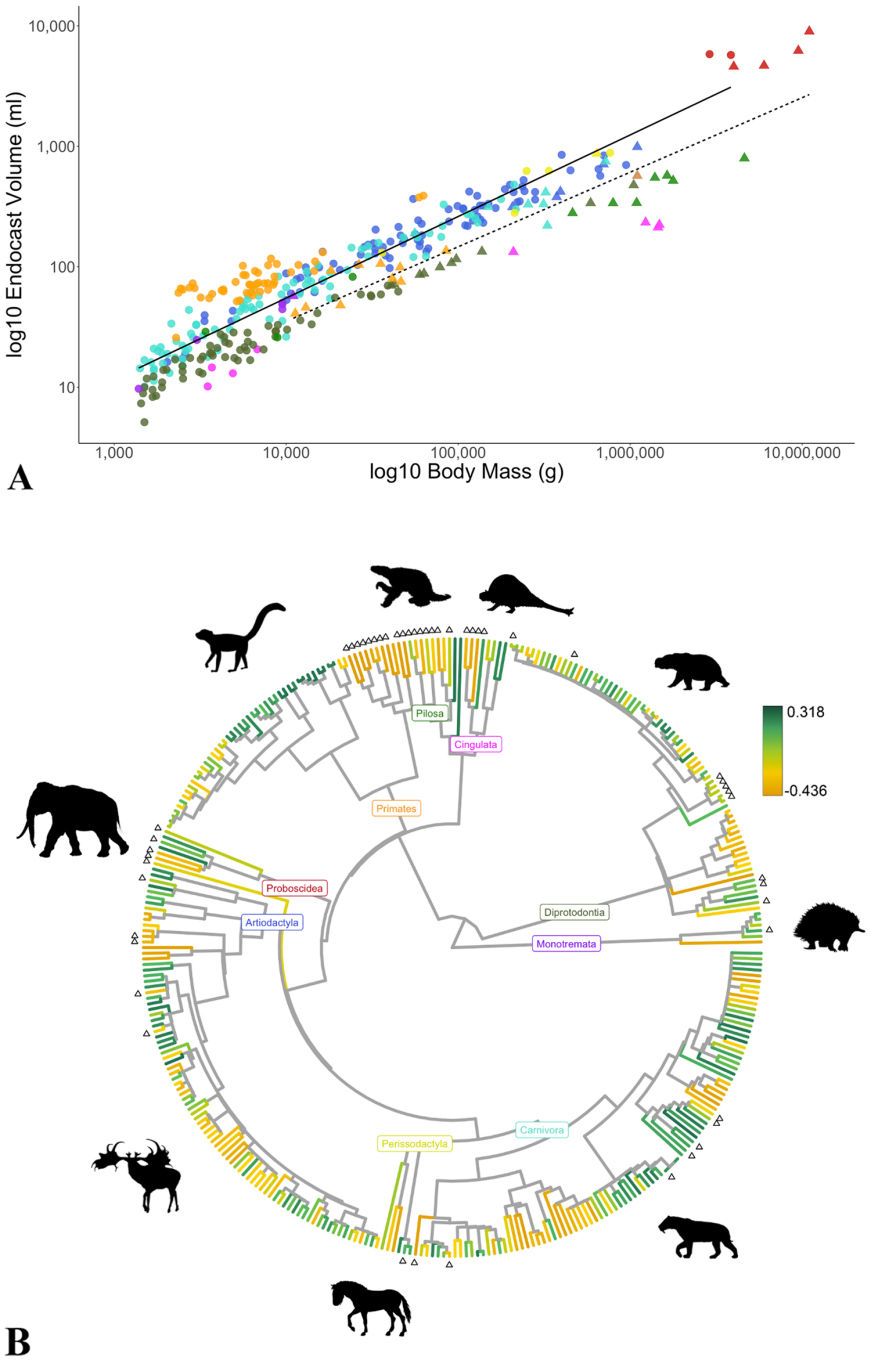
Brain size corrected for body size in extinct vs. extant species. (**A**) Log10 transformed endocast volume as a function of body mass, extinction status (extant: circles and filled regression line; extinct: triangles and dashed regression line) and taxonomic order. Blue: Artiodactyla; Turquoise: Carnivora; Pink: Cingulata; Olive green: Diprotodontia; Purple: Monotremata; Brown: Notoungulata; Yellow: Perissodactyla; Green: Pilosa; Orange: Primates; Red: Proboscidea. (**B**) The phylogenetic tree used in the analyses. Terminal branches are colored according to residuals of endocast volume versus body mass regression with order as a random effect (large brains: green; small brains: gold). Triangles at tips indicate extinct species. Animal silhouettes were available under Public Domain license at phylopic (http://phylopic.org/), unless otherwise indicated. Specifically, clockwise starting from the top, *Doedicurus* (http://phylopic.org/image/d4c04486-a358-4b04-a4a7-39b8b3c74630/); *Diprotodon* (http://phylopic.org/image/d72a4bfb-6bef-417a-bd58-9ed7751317c5/) available for reuse under the creative commons attribution 3.0 Unported (https://creativecommons.org/licenses/by/3.0/) image by Dmitry Bogdanov (vectorized by T. Michael Keesey); *Tachyglossus* (http://phylopic.org/image/6885c062-5deb-4ebf-a481-752186819108/); *Smilodon* (http://phylopic.org/image/6546f44e-3d4a-4dcb-bdda-934c40b22848/) available for reuse under the creative commons attribution 3.0 Unported (http://creativecommons.org/licenses/by-sa/3.0/) image by Matt Martyniuk (vectorized by T. Michael Keesey); *Equus* (http://phylopic.org/image/85d95128-912c-427a-9542-138e1dbf5651/) available for reuse under the creative commons attribution 3.0 Unported (https://creativecommons.org/licenses/by/3.0/) image by Mercedes Yrayzoz (vectorized by T. Michael Keesey); *Megaloceros* (http://phylopic.org/image/a85b378b-2287-4cf1-a0f1-0ad53cd56f56/); *Palaeoloxodon* (http://phylopic.org/image/e6beff81-167d-4882-b86f-24ef9a405835/); lemuriformes (http://phylopic.org/image/eefe8b60-9a26-46ed-a144-67f4ac885267/), available for reuse under Attribution-ShareAlike 3.0 Unported (https://creativecommons.org/licenses/by-sa/3.0/) image by Smokeybjb; *Nothrotheriops* (http://phylopic.org/image/6d0c872b-1d01-4d4d-a4ba-3448aebfadcf/).

In a linear regression model, extant species had endocast volumes 83% larger, on average, than similar-sized extinct counterparts, 53% larger when order was used as a random effect, and 14% when correcting for phylogeny (Fig. 1, Table 1). A pgls on the scaled tree had the lowest AIC value (− 691.01) and found that extinction status was a significant predictor (p = 0.008) and that extant species had significantly larger brains per their different intercepts (extant species intercept = − 0.16, extinct species intercept = − 0.21). All models that used extinction status as a predictor performed better than those that did not (per their AIC score) showing a significant difference in intercept between extinct and extant species (Table 1).

**Table 1:** Linear regression models of (log10 transformed) endocast volume as a function of extinction status, body size, order (as random effect), and phylogeny. Ratio: endocast volume of extant species divided by that of extinct species.

Predictor	All species intercept	Extant species intercept	Extinct species intercept	Ratio	Extinction status p-value	Model R^2^	AIC
**Linear mixed effect regressions**							
Body mass	− 0.71	NA	NA	NA	NA	0.86	− 81.24
Body mass + extinction status	NA	− 0.93	− 1.19	183%	< 0.001	0.87	− 128.76
Body mass + order	− 0.57	NA	NA	NA	NA	0.95	− 422.76
Body mass + extinction status + order	NA	− 0.77	− 0.96	153%	< 0.001	0.96	− 479.45
**Phylogenetic least square regressions**							
Body mass + phylogeny	− 0.54	NA	NA	NA	NA	0.98	− 684.93
Body mass + extinction status + phylogeny	NA	− 0.60	− 0.66	114%	< 0.001	0.98	− 688.53
Body mass + phylogeny (rescaled tree)	− 0.097	NA	NA	NA	NA	NA	− 685.89
Body mass + extinction status + phylogeny (rescaled tree)	NA	− 0.16	− 0.21	114%	0.008	NA	− 691.01

A pglmm model with extinction status as the response, showed that endocast volume (slope = − 10.73 ± 3.03) and body mass (slope = 10.49 ± 2.17), explained which species survived and which went extinct very well (for both predictors p < 0.001; model R^2^ = 0.84). Body mass alone (p < 0.001; model R^2^ = 0.81) was also a good predictor of extinction, but the model including brain size had a much lower AIC score (135.72 vs. 178.03). This relationship held true across all pglmm models. Models that corrected for evolutionary rate variation performed better than the BM model, confirming that extant species had larger brains than extinct species, and that brain size was a significant predictor of extinction status (Table 2). Rates of brain size evolution (body mass corrected) do not differ between extinct and extant species (p = 0.45, and p = 0.64 when body size and brain volume data are from the same individual).

**Table 2:** Pglmm models. Extinction status as a function of endocast volume, body size, order (as random effect), and phylogeny on three trees; Brownian motion, rescaled with body size, rescaled with brain size. All p-values are ≤ 0.001.

Tree	Predictors	Endocast volume slope	Body mass slope	AIC	R^2^
Not rescaled	Phylogeny + body mass	NA	3.74	178.03	0.81
	Phylogeny + body mass + endocast volume	− 10.73	10.49	135.72	0.84
	Phylogeny + body mass + endocast volume + order	− 11.33	11.32	133.36	0.82
Rescaled, RRphylo with body size as a predictor	Phylogeny + body mass	NA	3.55	175.65	0.82
	Phylogeny + body mass + endocast volume	− 10.13	9.73	134.03	0.84
	Phylogeny + body mass + endocast volume + order	− 11.28	11.33	132.41	0.82
Rescaled, RRphylo on brain size only	Phylogeny + body mass	NA	3.74	165.57	0.83
	Phylogeny + body mass + endocast volume	− 10.56	10.12	131.83	0.85
	Phylogeny + body mass + endocast volume + order	− 12.05	11.86	123.11	0.82

## Discussion

We found that extinction probability increased as body size increased and decreased as brain size increased. Models using brain size and body size explain extinction status better than models using body size alone. In keeping with this, we found that extant mammals have larger brains than their similar-sized extinct relatives, suggesting that large brain size was an important factor in explaining Late Quaternary extinctions.

Extant species with relatively larger brains are better adapted to face novel and anthropogenic threats and large brains characterize invasive species^23–25,28^. In extant birds^39,40^ and mammals^41^ large brains are associated with lower extinction risk. Our results suggest that the large brains of species that survived the Late Quaternary equipped them with an enhanced ability to cope with modern anthropogenic pressures and the contemporaneous effect of intense climate change^23,29,42^. A potential effect of brain size on life history, whereby larger brains were associated with slower maturation and reproductive rate, enhancing extinction risk, was not supported due to the selective survival of large-brained species.

The absence of significant rate difference in the evolution of brain volume between extinct and extant species indicates that developing a large brain was probably not an adaptive response to Late Quaternary perturbations (i.e., species did not evolve large brains in response to contemporary conditions). Rather a comparatively small brain made some of the megafauna more vulnerable to extinction. Since large brains evolved for reasons unrelated to those that promoted their survival during the Late Quaternary (such as changes in climate, predation regime, life history, changes in body size and sociality^21,22,43^), i.e., our data suggest that they worked as an exaptation^44^. This may explain why some large mammals went extinct (e.g., the relatively small brained *Mammut americanum*) while others, such as the large brained extant elephants (*Elephas maximus* and *Loxodonta africana*), survived.

Extinct species from the orders Notoungulata, Pilosa, Cingulata, and Diprotodontia possessed, on average, the smallest brains (relative to those predicted for mammals of similar sizes) of any species in our dataset (Fig. 1). This may be one of the reasons why almost no large-bodied species of these orders have survived the Pleistocene, since extinction probability increased with body size and decreased with increasing brain size. The largest extinct and extant diprotodontians in our database are *Diprotodon optatum* (~ 1 tonne) and *Macropus rufus* (~ 45 kg), respectively (Appendix 2)—a 22-fold difference. Even more extreme is the difference between the largest extinct and extant pilosans, *Lestodon armatus* (~ 4.6 tonnes) and *Myrmecophaga tridactyla* (~ 24 kg), and cingulatans, *Glyptodon reticulatus* (~ 1.5 tonnes) and *Euphractus sexcinctus* (~ 7 kg): 192-fold and 214-fold differences, respectively. In the large-brained orders Proboscidea and Carnivora, on the other hand, the largest extinct species in our database are *Palaeoloxodon antiquus* (11 tonnes) and *Arctodus simus* (720 kg), respectively while the largest extant species are *Loxodonta africana* (~ 3.9 tonnes) and *Ursus maritimus* (~ 211 kg)—2.8 and 3.4-fold differences, respectively (Appendix 2). Members of small-brained orders may not have possessed the behavioral flexibility needed to cope with a changing climate and/or the arrival of *Homo sapiens*^5,45^. Diprotodonts and xenarthrans inhabit South America and Australia, the continents that were most heavily hit by the Late Quaternary extinction wave^7,16^. It is hypothesized that these continents suffered significantly more extinctions due to more severe climate change and complete naivety to human hunters^45,46^. We proposed that these factors coupled with small brain size left these taxa more vulnerable to the changing conditions of the Late Quaternary and help explain the disproportionate number of extinctions on these continents.

We conclude that large-brained species had better chances of surviving the Late Quaternary. Although body mass was clearly the most important factor, large brains were selected for: brain size helps explain which large species were most likely to survive to the present day. Large-brained species were likely better adapted to a rapidly changing climate and/or the pressures imposed by the most dangerous of novel predators, man.

## Materials and methods

Since brains do not fossilize, we used endocast volumes as an index for brain size. The endocast is a measure of the volume of the cranial cavity, and is a reliable proxy for brain size in mammals^22^. We recorded endocast volumes from the literature and directly from skull digital models, using the recently implemented *endomaker* function in the R package Arothron^47^. *Endomaker* was found to be at least as accurate as other available software for endocast volume calculation, and provides estimates nearly identical to manual methods^47^_._ We recorded body masses and extinction dates from the literature. We used body mass (calculated with linear measurements using allometric equations and recorded from the literature^48^), and endocast volumes, calculated from the same specimens if these were available, to ensure that the relationship between body mass and endocast volume is not confounded by age, sex, and geographic variation. We constructed a database for all mammals that went extinct between the beginning of the last Ice Age, at some 115 ka^49,50^ and the first historical extinctions (~ 500 years ago, i.e. until the period covered by the IUCN to depict modern extinctions) for which we had endocast size data. The latest dates set to exclude historic extinctions, which are possibly related to activities of humans with modern technology. We restricted the dataset to the taxonomic orders that contain species that went extinct in the Pleistocene, and to a roughly similar range of body sizes. The smallest extinct species in our dataset is the monotreme *Megalibgwilia ramsayi* (11 kg^51^). We therefore excluded all mammals smaller than 1.4 kg (e.g., all bats), the size of the smallest member of the taxonomic order of *M*. *ramsayi*, the extant Platypus (*Ornithorhynchus anatinus*). The domestic camelids, *Camelus dromedarius* and *C. bactrianus*, are the only domestic species in our dataset. Brains of domestic animals are thought to be smaller than those of their wild kin, and thus the use of extant (presumably small-brained) domestic species makes our analyses conservative^52,53^. We excluded birds and reptiles from this study because few such taxa went extinct during the period we study, only a small fraction of which have endocast volume data. Furthermore, reptile and bird extinctions have widely been attributed to insularity (and flightlessness in birds)^54,55^, whereas mammal extinctions during that period were widespread on continents^16^. We only included species from land masses larger than 50,000 km^2^ (following^56^) because of potential effects of insularity on brain size^52,57^, and extinction probability^58–61^.

When brain mass data were available for an extant species, and endocast volume was not, we first converted brain mass to brain volume by dividing it by 1.036^62^, then converted brain volume to endocast volume using the formula: log Endocast Volume = − 0.0015 + 1.0222 (log brain volume)^63,64^. When both brain mass and endocast volume were available we only used endocast volume, which provides direct comparison with extinct species.

### Analyses

We applied linear regression models in order to test if extant species have larger or smaller brains than extinct species by using body size and extinction status as predictors and endocast volume as the response. Body masses, and endocast volumes, were log10-transformed before analyses. We corrected for phylogenetic effects in relative brain size by applying multiple different approaches^65^. We first accounted for phylogeny by using mixed effect models with taxonomic orders as random effects using R packages lme4 and lmerTest^66,67^. These non-phylogenetic analyses were also added because extinction status is not necessarily a derived trait explained by relationship to other species.

We then assembled a tree for all species we had the data for based on^52,68^ and additional sources (see Appendix 3). We first tested whether brain size changes per extinction status while accounting for phylogeny, by means of phylogenetic least square regression (pgls). Under pgls, brain size evolution is assumed to proceed according to the Brownian motion (BM) model of evolution. This assumption is possibly invalid in mammals, since major differences in brain mass scaling are known to occur among mammalian orders^21,69^. To account for this, we used a model-free phylogenetic comparative method using the *RRphylo* package in R^37^. Under *RRphylo* the rates of brain size changes across the tree are estimated by means of phylogenetic ridge regression, minimizing rate variation within clades by means of maximum likelihood estimation. This procedure effectively accounts for differences in the regime of brain size evolution which may accrue to different parts of the tree. The *RRphylo* rates were used to rescale the tree branch lengths while holding constant the total evolutionary time represented by the tree. We used the RRphylo package function *PGLS_fossil* to perform pgls by using the original tree (i.e., assuming the Brownian motion model of evolution) and on the tree rescaled according to *RRphylo* rates computed on brain size evolution, regressing brain size versus body size and extinction status.

We tested whether endocast volume is a significant predictor of extinction status under an explicit phylogenetic context by Phylogenetic Generalized Linear Mixed Model regression (pglmm^36^) using the R package phyr^70^. In pglmm, the phylogenetic covariance matrix is derived from the tree and added to the regression as a random factor to account for phylogenetic effects. Overall, we ran nine pglmm models. In all models the response variable was the extinction status (i.e. whether the species has gone extinct during the late Quaternary or not). We used (a) body size alone, (b) brain size and body size, and (c) brain size, body size and taxonomic order, as predictors (the first two as fixed effects, order as a random effect). To include phylogenetic effects, we used (1) the tree with untransformed branch lengths (that is equivalent to assuming Brownian motion); (2) the tree where branch lengths were transformed according to rates of brain size evolution alone, and (3) the tree where branch lengths were transformed according to brain size evolution while accounting for body size. This last option (3) is possible by implementing a second *RRphylo* analysis, where body size is used as a predictor of brain size evolution^71,72^. The models with order as a random effect were used because controlling for the tree phylogenetic variance covariance matrix assumes the same scaling relationship between brain and body size across the tree, yet major differences among orders were found to occur in mammals^57^.

Comparing extant versus extinct species brain size, one potential bias would be introduced if brain size evolution started to accelerate after the Late Pleistocene extinction. Since *RRphylo* rates are regression rates between two consecutive phenotypes aligned along the phylogeny^37^, this would imply the brain size evolutionary rates at the branches leading to extant species would be higher than those leading to extinct ones. We tested this using the *search.shift* function in RRphylo^37^. This function tests, by means of randomization, whether the average absolute rate of brain volume evolution computed for extinct and extant species differ. We performed *search.shift* on the rates of brain size evolution computed accounting for body size as a predictor of brain size.

Data were analysed in R and organized and graphed using the tidyverse package^73^. We compared models using Akaike information criterion (AIC) values using the MuMin package^74^. We performed a sensitivity analysis by excluding all species for which only mean body mass (rather than body mass calculated from the same specimen from which endocast volumes were obtained) were available (Supplementary material).

## Supplementary Information

Supplementary Information 1.

Supplementary Information 2.

Supplementary Information 3.

Supplementary Information 4.

Supplementary Information 5.

Supplementary Information 6.

Supplementary Information 7.

## Data Availability

All data are available in the main text or the supplementary materials.
