# Supplementary material for: Small brains predisposed Late Quaternary mammals to extinction
Source: Sci Rep. 2022 Mar 31;12:3453. doi: 10.1038/s41598-022-07327-9 (PMC8971383; doi:10.1038/s41598-022-07327-9)
Supplement: Supplementary file 6 — Supplementary Information 6. [file 41598_2022_7327_MOESM6_ESM.pdf]

Small brains predisposed Late Quaternary mammals to extinction

Jacob Dembitzer[1]*, Silvia Castiglione[2], Pasquale Raia[2] and Shai Meiri[1,3]

[1]School of Zoology, Tel Aviv University, Tel Aviv, 6997801, Israel

[2]Dipartimento di Scienze della Terra, dell'Ambiente e delle Risorse, Università di Napoli Federico II, 80126, Napoli, Italy

[3]The Steinhardt Museum of Natural History, Tel Aviv University, Tel Aviv, 6997801, Israel

*Corresponding author. Email: jacobd@mail.tau.ac.il

Sensitivity Analysis

We analyzed whether the use of mean (rather than individual) body size estimates affected our results by analyzing the 'same specimen' dataset. This dataset excludes 18 species from the full dataset, 13 of which are extinct. Their removal therefore greatly restricts the number of extinct species (37 species left) and skews the extant/extinct species ratio. We therefore randomly eliminated 71 extant species and re-run the analyses, in order to maintain an equal ratio of extinct to extant taxa (17.2 %). We repeated this procedure 100 times. At each iteration, we ran a pglmm (including orders and phylogeny as random effects and extinction status as the response variable) and a pgls model (including brain volume as the response variable, body size and extinction status as the explanatory variables) and calculated the AIC of the models with unscaled and RRphylo-rescaled trees. The model with the lowest AIC score at each iteration was chosen and inspected in order to count the number of significant results across the 100 iterations.

It has been proposed that using mean mass of marsupials is preferential to mass measured from specimens to avoid underestimations[1]. Therefore, we analyzed our data with these updated masses to ensure that our results were not biased. Moreover, Smaers et al.[2] published a large dataset of over 1000 species. We note that all relevant Late Quaternary species from this study were present in our dataset. Many of the extant species however did not meet our criteria for inclusion in our study (e.g., brain and body size measured from the same specimen, >1.4kg, it is not known whether a species is from a small island, and it is not known if brain sizes were converted from endocast volumes). We added species from this study that met our criteria of body size and order regardless of whether or not other criteria were met and analyzed the dataset.

Results

In the one hundred pglmm iterations including order and phylogeny as random effects, the model using the tree rescaled according to RRphylo always produced lower AIC scores than models with unscaled trees. Out of the 100 best models, 98 were run with RRphylo rescaled according to brain size, and two with RRphylo rescaled for brain size with body size as a predictor. All 100 pglmm models indicate a significant and negative effect of brain size on extinction (i.e., larger brain, lower likelihood to be classified as extinct). The random effect 'order' is significant 98 times, indicating that there are major differences between orders.

In the 100 pgls iterations, brain size was found to be significantly smaller in extinct species 71 times (56 times when the best model was found to be the pgls using the unscaled tree, 15 times by using the RRphylo-rescaled tree). This result is likely the product of removing entire clades of small-brained extinct species (glyptodonts, machairodont cats) erasing much of the clade-level differences in brain size across the mammalian tree (and greatly reducing statistical power). The number of significant results in 100 model runs expected by chance is 5 and so our sensitivity analyses support the hypothesis that extant species have larger brains than extinct species.

The dataset with updated masses for marsupials showed that in a normal linear regression model extant species had brains that were 81% larger than extinct species, in a linear mixed model 52%, and in a phylogenetically informed regression 17%. The results are nearly identical to those presented in our main analysis. Moreover, when including data from Smaers et al. 2021, a normal linear regression showed that extant species had brains that were 64% larger than their extinct counterparts and a linear mixed model showed that they had brains that were 38% larger. All additional data are presented in appendices.

1. Weisbecker, V., Ashwell, K. & Fisher, D. An improved body mass dataset for the study of marsupial brain size evolution. *Brain. Behav. Evol.* **82**, 81 (2013).
2. Smaers, J. B. *et al.* The evolution of mammalian brain size. *Sci. Adv.* **7**, 1–12 (2021).
